# Antibacterial and anti-biofilm activities of probiotic *Lactobacillus plantarum* against *Listeria monocytogenes* isolated from milk, chicken and pregnant women

**DOI:** 10.3389/fmicb.2023.1201201

**Published:** 2023-07-19

**Authors:** Rasha M. M. Abou Elez, Ibrahim Elsohaby, Abdul-Raouf Al-Mohammadi, Marwa Seliem, Asmaa B. M. B. Tahoun, Amira I. Abousaty, Reem M. Algendy, Eman A. A. Mohamed, Nashwa El-Gazzar

**Affiliations:** ^1^Department of Zoonoses, Faculty of Veterinary Medicine, Zagazig University, Zagazig, Egypt; ^2^Department of Infectious Diseases and Public Health, Jockey Club College of Veterinary Medicine and Life Sciences, City University of Hong Kong, Kowloon, Hong Kong SAR, China; ^3^Centre for Applied One Health Research and Policy Advice (OHRP), City University of Hong Kong, Kowloon, Hong Kong SAR, China; ^4^Department of Animal Medicine, Faculty of Veterinary Medicine, Zagazig University, Zagazig, Egypt; ^5^Department of Science, King Khalid Military Academy, Riyadh, Saudi Arabia; ^6^Department of Food Hygiene, Safety and Technology, Faculty of Veterinary Medicine, Zagazig University, Zagazig, Egypt; ^7^Department of Botany and Microbiology, Faculty of Science, Zagazig University, Zagazig, Egypt; ^8^Department of Microbiology, Faculty of Veterinary Medicine, Zagazig University, Zagazig, Egypt

**Keywords:** *Listeria monocytogenes*, virulotyping, serotyping, antimicrobial resistance, biofilm, *Lactobacillus plantarum*

## Abstract

*Listeria monocytogenes* (*L. monocytogenes*) is a foodborne pathogen that poses significant risks to public health and food safety. The present study aimed to identify the presence of *Listeria* spp. in various samples, including pasteurized milk, chicken fillets, and stool samples from pregnant women in Sharkia Governorate, Egypt. Additionally, the study identified the serotypes, virulence-associated genes, antimicrobial resistance patterns, and biofilm formation in *L. monocytogenes* isolates. Moreover, the antibacterial and anti-biofilm activity of *Lactobacillus plantarum* ATCC 14917 (*L. plantarum*) against *L. monocytogenes* isolates was investigated. A cross-sectional study was conducted from August 2021 to January 2022 to collect 300 samples of pasteurized milk, chicken fillets, and stool from pregnant women admitted to outpatient clinics of hospitals. The results showed that 32.7% of the samples were positive for *Listeria* spp., including *L. innocua* (48.9%), *L. monocytogenes* (26.5%), *L. ivanovii* (14.3%), *L. grayi* (5.1%), and *L. welshimeri* (5.1%). Among all *L. monocytogenes* isolates, *hlyA*, *actA*, *inlC*, and *inlJ* virulence-associated genes were detected. However, the virulence genes *plcB*, *iap*, and *inlA* were found in 10 (38.5%), 8 (30.8%), and 25 (96.2%) isolates, respectively. The *L. monocytogenes* isolates classified into four serotypes (1/2a, 1/2b, 1/2c, and 4b), with 1/2a and 4b each identified in 30.8% of the isolates, while 1/2b and 1/2c were identified in 19.2% of the isolates. All *L. monocytogenes* isolates showed 100% resistance to streptomycin, kanamycin, and nalidix acid, and 92.3% of isolates showed gentamicin resistance. However, all isolates were susceptible to ampicillin and ampicillin/sulbactam. Multidrug resistance (MDR) was observed in 20 (76.9%) *L. monocytogenes* isolates. The biofilm formation ability of 26 *L. monocytogenes* isolates was evaluated at different incubation temperatures. At 4°C, 25°C, and 37°C, 53.8, 69.2, and 80.8% of the isolates, respectively, were biofilm producers. Furthermore, 23.1% were strong biofilm producers at both 4°C and 25°C, while 34.6% were strong biofilm formers at 37°C. Treating *L. monocytogenes* isolates with *L. plantarum* cell-free supernatant (CFS) reduced the number of biofilm-producing isolates to 15.4, 42.3, and 53.8% at 4°C, 25°C, and 37°C, respectively. *L. plantarum’s* CFS antibacterial activity was tested against six virulent, MDR, and biofilm-forming *L. monocytogenes* isolates. At a concentration of 5 μg/mL of *L. plantarum* CFS, none of the *L. monocytogenes* isolates exhibited an inhibition zone. However, an inhibition zone was observed against *L. monocytogenes* strains isolated from pasteurized milk and pregnant women’s stools when using a concentration of 10 μg/mL. Transmission electron microscopy (TEM) revealed that *L. plantarum* CFS induced morphological and intracellular structural changes in *L. monocytogenes*. In conclusion, this study identified virulent MDR *L. monocytogenes* isolates with strong biofilm-forming abilities in food products in Egypt, posing significant risks to food safety. Monitoring the prevalence and antimicrobial resistance profile of *L. monocytogenes* in dairy and meat products is crucial to enhance their safety. Although *L. plantarum* CFS showed potential antibacterial and anti-biofilm effects against *L. monocytogenes* isolates, further research is needed to explore its full probiotic potential.

## 1. Introduction

The genus *Listeria* is a Gram-positive, non-spore-forming, facultative anaerobic, rod-shaped bacteria (Orsi et al., 2011). It comprises several species, including *Listeria monocytogenes* (*L. monocytogenes*), *L. innocua*, *L. ivanovii*, *L. welshimeri*, *L. grayi*, *L. seeligeri*, as well as the newly discovered *L. marthii*, *L. weihenstephanensis*, *L. rocourtiae*, and *L. fleischmannii* (Hellberg et al., 2013). *Listeria* spp. are found in various environmental sources such as soil, water, food, and the feces of humans and animals (Zunabovic et al., 2011). Furthermore, it can grow at low temperatures, high salt concentrations, and a wide pH range (Walker et al., 1990).

*Listeria monocytogenes* is the most pathogenic species among *Listeria*, causing a highly fatal opportunistic foodborne infection known as listeriosis (Vázquez-Boland et al., 2001). This disease predominantly affects pregnant women, elderly, neonates, and immunocompromised or debilitated patients, although it can also develop in healthy individuals. It causes mortality of 30% in immunosuppressed individuals, elderly and neonate worldwide (Drevets and Bronze, 2008). Human listeriosis in developing countries is mostly acquired through consumption of contaminated milk products including soft cheeses, meat products including ready-to-eat meat products (Scallan et al., 2011) and raw, smoked or cured fish products and seafood (Jami et al., 2014).

*Listeria monocytogenes* has thirteen serotypes based on somatic (O) and flagellar (H) antigen reactions (Meloni, 2014). Serotypes 4b, 1/2a, and 1/2b are predominant in human infections (Zeinali et al., 2015). The pathogenicity of *L. monocytogenes* is attributed to variant virulence factors, such as internalin (encoded by *inlA*, *inlB*, *inlC*, and *inlJ* genes), listeriolysin O (encoded by *hly* gene), phosphatidylinositol phospholipase C (encoded by *plcA*), actin polymerization protein (encoded by *actA*), and invasive associated protein (*iap*), as well as the regulatory system for gene expression of virulence (*PrfA*; Liu et al., 2007).

*Listeria monocytogenes* are typically sensitive to commonly used antibiotics, but multiple drug-resistant strains have been found in cases of human listeriosis, food and environment (Moreno et al., 2014; Noll et al., 2017; Tahoun et al., 2017). The excessive use of antibiotics as growth promoters has hastened the evolution of *L. monocytogenes* toward resistance (Doyle et al., 2013), in keeping with the global trend of rising antibiotic resistance among foodborne pathogens (Harakeh et al., 2009). Biofilms help bacteria resist environmental stressors like dehydration and antimicrobial treatments. *L. monocytogenes* has the ability to form biofilms on various surfaces in food processing environments, including equipment, utensils, and food contact surfaces, which contributes to its survival and pathogenicity (Møretrø and Langsrud, 2004; Folsom et al., 2006). To effectively eradicate *L. monocytogenes* biofilms, several strategies and interventions can be employed, including the use of chemical disinfectants, mechanical removal, biofilm-disrupting agents, heat treatment, and radiation (Fagerlund et al., 2020).

To address the limited treatment options for *L. monocytogenes*, various alternative therapies have been explored to inhibit its growth in foods due to the rapid development of antimicrobial resistance in this pathogen (Matle et al., 2020). One approach is the use of lactic acid bacteria (LAB) strains that produce probiotics with antimicrobial properties. Probiotics have been shown to reduce the growth, adhesion, and biofilm formation of *L. monocytogenes* (Merino et al., 2019) while enhancing host immunity (Dhama et al., 2015). Therefore, the objectives of this study were to (i) detect *Listeria* spp. in pasteurized milk, chicken fillets, and stool samples from pregnant women in Sharkia Governorate, Egypt, (ii) identify the serotypes, virulence-associated genes, antimicrobial resistance patterns and biofilm formation in *L. monocytogenes* isolates, and (iii) investigate the antibacterial and anti-biofilm effect of *Lactobacillus plantarum* (*L. plantarum*) on *L. monocytogenes* isolates.

## 2. Materials and methods

### 2.1. Sample collection

A cross-sectional study carried out between August 2021 and January 2022 to collect pasteurized milk and chicken fillets samples (*n* = 100, each) from various retail markets across Sharkia Governorate, Egypt. Simultaneously, 100 stool samples were collected from pregnant women admitted to outpatient clinics of hospitals located in the same localities as the retail markets. The collected samples were labeled with retail market/hospital ID, location, date, and time of sampling before being sent to the laboratory for later analysis.

### 2.2. Isolation and identification of *Listeria* spp.

The US Food and Drug Administration (FDA) protocol (Hitchins et al., 2017) was used to isolate and identify *Listeria* spp. from pasteurized milk, chicken fillet, and stool samples. Initially, samples were pre-enriched in buffered peptone water (Himedia Lab, Mumbai, India) and incubated for 48 h at 37°C. Next, 25 mL of each pre-enriched sample were added to 225 mL of *Listeria* enrichment broth (Himedia Lab, Mumbai, India) and incubated for 48 h at 30°C. A loopful from the enriched broth was directly streaked onto Agar *Listeria* Ottaviani Agosti (ALOA) (Himedia Lab, Mumbai, India) and incubated for 24–48 h at 37°C. Colonies (Walker et al., 1990; Zunabovic et al., 2011) exhibiting typical morphologically were cultured on tryptic soy agar yeast extract (TSAye) at 35°C for 24–48 h. The colonies were presumptively identified using Gram staining, biochemical tests including haemolytic, catalase, and oxidase activities, as well as rhamnose, xylose, and mannitol fermentation (Mac Faddin, 2000). Additionally, the Oxoid *Listeria* Test Kit (Oxoid, UK) was used for Latex Agglutination Test.

### 2.3. Molecular characterization of *Listeria monocytogenes*

Genomic DNA was extracted from *L. monocytogenes* isolates using the QIAamp DNA Mini kit (QIAGEN GmbH, Hilden, Germany). A PCR amplification of 553 bp was performed on the extracted DNA using primers (Supplementary Table S1) specific for 16S rRNA (Lantz et al., 1994).

*Listeria monocytogenes* confirmed isolates were identified for virulence-associated genes using primers targeting *hly*, *actA*, *plcB*, *iap* (Cao et al., 2018), and *inlA*, *inlC*, *inlJ* genes (Liu et al., 2007; Supplementary Table S1). Additionally, the serotypes of *L. monocytogenes* isolates were determined using four genes: Imo0737 (619 bp), lmo1118 (906 bp), ORF2819 (417 bp), and ORF2110 (597 bp) (Doumith et al., 2004). Positive controls, including reference strains CDC F4555 (4b), ATCC 19111 (1/2a), CDC F4976 (1/2b), and ATCC 19112 (1/2c), were used in the PCR assay alongside the tested isolates.

### 2.4. Antimicrobial susceptibility test

The antibiotic susceptibility of the confirmed *L. monocytogenes* isolates was determined using the disk diffusion method (Oxoid, UK), following Clinical and Laboratory Standards Institute (CLSI) guidelines (Clinical and Laboratory Standards Institute, 2018). The antibiotic disks tested were ampicillin (AMP, 10 μg/mL), ampicillin/sulbactam (SAM, 20 μg/mL), amoxicillin-clavulanate (AMC, 30 μg/mL), cefotaxime (CTX, 30 μg/mL), cephalothin (CEF, 30 μg/mL), gentamicin (GEN, 10 μg/mL), streptomycin (STR, 10 μg/mL), tetracycline (TET, 30 μg/mL), kanamycin (KAN, 30 μg/mL), ciprofloxacin (CIP, 5 μg/mL), nalidix acid (NA, 30 μg/mL), trimethoprim-sulfamethoxazole (SXT, 25 μg/mL), chloramphenicol (CHL, 30 μg/mL), erythromycin (ERY, 15 μg/mL), and meropenem (MEM, 10 μg/mL). Mueller-Hinton agar plates were used (Oxoid, UK) with incubation at 35°C for 24 h, then the zone of inhibition was recorded as resistant (R), intermediate (I), or sensitive (S) according to CLSI standards. Isolates that exhibited resistance to three or more distinct antimicrobial classes were categorized as being multidrug resistant (MDR), as defined by Magiorakos, Srinivasan (Magiorakos et al., 2012). Additionally, the multiple antibiotic resistances (MAR) index was calculated for all isolates by applying the formula a/b, where “a” represents the number of antimicrobials to which an isolate was resistant and “b” represents the total number of antimicrobials tested. This calculation was carried out in accordance with the protocol specified by Krumperman (1983).

### 2.5. Biofilm formation and quantification

The biofilm forming ability of *L. monocytogenes* isolates was evaluated at various storage temperature (4°C, 25°C, and 37°C) using the microtiter plate assay (Kırmusaoğlu, 2019). A bacterial suspension was prepared in Mueller Hinton broth and adjusted to 0.5 McFarland (1.5 × 10^8^ CFU/mL). A 100 μL of the bacterial suspension was inoculated into each of the three sterile polystyrene microtiter plates in triplicate. The plates were incubated at 4°C, 25°C, and 37°C for 24 h (plate I, II, and III, respectively). In each plate, three wells containing only broth were left as negative controls. To remove free-floating cells, the media from the plate wells were discarded and washed twice with 0.2 mL of phosphate-buffered saline (PBS, pH 7.2). The plates were inverted, and PBS was removed by blotting with paper towels. The biofilm was fixed by adding 150 μL of ethanol for 20 min, and the cells adhered to the microtiter plates were stained with 150 μL of crystal violet for 15 min at room temperature after removing the stain. The wells were washed twice with PBS and air dried for 1 h.

Biofilm quantification was carried out by adding 150 μL of 95% ethanol to each well for 45 min. The optical density (OD) was then measured at a wavelength of 570 nm (OD_570_) using an ELISA reader (Sunrise, Tecan) after adjusting to the negative control (OD_NC_) at zero. Mean and standard deviation of OD values were recorded for all *L. monocytogenes* isolates and negative controls. The isolates were then classified as negative (OD_570_ ≤ OD_NC_), weak (OD_NC_ < OD_570_ ≤ 2 x OD_NC_), moderate (2 × OD_NC_ < OD_570_ ≤ 4 × OD_NC_), or strong (4 × OD_NC_ < OD_570_) biofilm formers (Saxena et al., 2014).

### 2.6. Effect of *Lactobacillus plantarum* on *Listeria monocytogenes*

The effect of *L. plantarum* ATCC 14917, a probiotic strain from the Belgian Co-ordinated Collection of Microorganisms (BCCM), on *L. monocytogenes* isolates recovered from pasteurized milk, chicken fillets, and pregnant women were evaluated.

#### 2.6.1. Extraction of *Lactobacillus plantarum* cell-free supernatant

*Lactobacillus plantarum* was cultured in de-Man, Rogosa, and Sharpe (MRS) broth for 18 h at 37°C, followed by centrifugation at 4000 rpm for 10 min at 4°C. The resulting supernatant was filter-sterilized using a 0.2 mm membrane syringe filter. The cell-free supernatant (CFS) of *L. plantarum* was collected to be used as an inhibitory agent against *L. monocytogenes* (Enan et al., 2014).

#### 2.6.2. Anti-biofilm activity of *Lactobacillus plantarum*

A bacterial suspension of *L. monocytogenes* co-cultured with *L. plantarum* CFS in Mueller Hinton broth, adjusted to 0.5 McFarland (1.5 × 10^8^ CFU/mL), and inoculated 100 μL of the suspension into microtiter plates as described above. The plates were then incubated at 4°C, 25°C, and 37°C for 24 h to assess the antibiofilm activity of *L. plantarum* at different incubation temperature.

To measure the extent of biofilm formation in the presence of *L. plantarum* CFS, we used an ELISA reader (Sunrise, Tecan) to measure the OD value of the biofilm at 570 nm, after adjusting to the negative control (OD_NC_) at zero. We calculated the percentage of biofilm inhibition using the following formula:


$$\begin{array}{l} Biofilminhibitionrate\;\left(\%\right)=\\ \;\;\;\;\;\;\;\;\;1-\frac{OD_{570}\;obtainedinpresence\;ofL.plantarum\;CFS\;}{OD_{570}\;inabsenceof\;L.plantarum\;CFS} \end{array}$$

The growth pattern of *L. monocytogenes* was evaluated in the presence and absence of *L. plantarum* CFS over a 24-h period in Mueller Hinton broth. The growth kinetics curve can be found in Supplementary Figure S1.

#### 2.6.3. Antibacterial activity of *Lactobacillus plantarum*

The antibacterial activity of *L. plantarum* CFS was assessed on six (two from each source) virulent, MDR and biofilm forming *L. monocytogenes* isolates using the agar well diffusion method (Osman et al., 2021). Briefly, 25 mL of melted Mueller Hinton agar was inoculated separately with 0.1 mL a 24-h-old bacterial suspension. The mixture was then poured into a Petri dish and left to solidify at room temperature for 30 min, then a 10 mm diameter well were made in the agar using a sterile cork borer. The wells were filled with 100 μL of the *L. plantarum* CFS. Positive and negative controls were implemented by filling wells with AMP (10 μg/mL) and broth media, respectively. The plates were then incubated at 37°C for 24 h, after which the inhibition zone diameters were measured using a ruler. Each isolate was tested in triplicate.

#### 2.6.4. Minimum inhibitory concentration assay

The minimum inhibitory concentration (MIC) value of *L. plantarum* CFS against *L. monocytogenes* isolates was determined using the agar well diffusion method as described above. *L. plantarum* CFS was prepared at various concentrations (10, 20, 30, 40, 50, 60, 70, 80, and 90 μg/mL) from the 100 μg/mL stock solution. Serial two-fold dilutions were made using sterile deionized water, with 0.05, 0.1, 0.15, 0.2, 0.25, 0.3, 0.35, 0.4, and 0.45 mL taken from the original stock, resulting in the respective concentrations mentioned. The dilutions were added to the wells and the plates were incubated at 37°C for 24 h. The mean and standard error of inhibition zone diameters were then determined, with all isolates tested in triplicate. The MIC was determined as the well containing the lowest concentration of *L. plantarum* CFS that still showed a zone of inhibition.

#### 2.6.5. Transmission electron microscope (TEM) analysis

*Lactobacillus plantarum* CFS was added to *L. monocytogenes* culture grown on nutrient broth media for 24 h (Amin, 2016). The bacterial cells were collected via centrifugation at 4000 rpm for 10 min, washed with distilled water, fixed with 3% glutaraldehyde, rinsed with phosphate buffer, and fixed again in a potassium permanganate solution for 5 min at room temperature. The samples were then dehydrated in an ethanol series (ranging from 10 to 90% for 15 min each), followed by absolute ethanol for 30 min. Samples were infiltrated with epoxy resin and acetone through a graded series, and ultimately in pure resin. Ultrathin sections were obtained on copper grids and double stained in uranyl acetate and lead citrate. The stained sections were then observed using TEM (JEOL JEM-1010, Tokyo, Japan). *L. monocytogenes* without *L. plantarum* CFS was used as a control.

### 2.7. Data analysis

The statistical analysis and data visualization were performed using R software (version 4.2.0). The Kruskal-Wallis test was used to assess the difference between the MAR index and sources of the samples, followed by multiple comparisons using the rank sums (Dunn test) with Bonferroni correction. A *p*-value of <0.05 was considered significant.

## 3. Results

### 3.1. *Listeria* spp. isolation and identification

Three hundred samples of pasteurized milk, chicken fillets, and stool from pregnant women were tested for *Listeria* spp. identification (Table 1). *Listeria* spp. were detected in 98 (32.7%) of the samples, with 10 (10%), 49 (52%), and 39 (23.1%) isolates retrieved from pasteurized milk, chicken fillets, and stool from pregnant women, respectively. Biochemical and *Listeria* Latex Agglutination test identified 48 (48.9%) *L. innocua*, 26 (26.5%) *L. monocytogenes*, 14 (14.3%) *L. ivanovii*, 5 (5.1%) *L. grayi*, and 5 (5.1%) *L. welshimeri* among the 98 *Listeria* spp. isolates (Table 1).

**Table 1:** Occurrence of *Listeria* spp. in pasteurized milk, chicken fillets and pregnant woman stools.

Type of samples	No. of samples analyzed	No. of *Listeria* spp. positive samples (%)	No. of species positive samples (%)				
			*L. monocytogenes*	*L. innocua*	*L. ivanovii*	*L. grayi*	*L. welshimeri*
Pasteurized milk	100	10 (10)	2 (20)	5 (50)	1 (10)	1 (10)	1 (10)
Chicken fillets	100	49 (52)	15 (30.6)	28 (57.1)	6 (12.2)	0 (0.0)	0 (0.0)
Pregnant women stool	100	39 (23.1)	9 (23.1)	15 (38.5)	7 (17.9)	4 (10.3)	4 (10.3)
Total	300	98 (32.7)	26 (26.5)	48 (48.9)	14 (14.3)	5 (5.1)	5 (5.1)

### 3.2. *Listeria monocytogenes* virulotyping and serotyping

All *L. monocytogenes* isolates had *hlyA*, *actA*, *inlC*, and *inlJ* virulence-associated genes. However, the *plcB*, *iap*, and *inlA* virulence-associated genes were found in 10 (38.5%), 8 (30.8%), and 25 (96.2%) isolates, respectively (Table 2). The recovered isolates belonged to four *L. monocytogenes* serotypes (1/2a, 1/2b, 1/2c, and 4b), with serotypes 1/2a and 4b each identified in 30.8% of the isolates, while 1/2b and 1/2c were identified in 19.2% of the isolates (Table 2).

**Table 2:** Serotypes, virulence genes, biofilm degree, and resistance patterns of *L. monocytogenes* isolates.

ID	Source	Serotypes	Virulence-associated genes^1^							Resistance patterns^2^	MAR index^3^	Biofilm degree^4^		
			*hlyA*	*actA*	*plcB*	*iap*	*inlA*	*inlC*	*inlJ*			4°C	25°C	37°C
M1	Milk	4b	+	+	+	+	+	+	+	AMC, CTX, CEF, GEN, STR, KAN, TET, NA, SXT, CHL, ERY, MEM	0.8	S	S	S
M2	Milk	4b	+	+	+	+	+	+	+	AMC, CTX, CEF, GEN, STR, KAN, TET, NA, SXT, CHL, ERY, MEM	0.8	S	S	S
M3	Milk	1/2a	+	+	−	+	+	+	+	GEN, STR, KAN, NA	0.27	N	W	W
M4	Milk	1/2b	+	+	−	−	+	+	+	CTX, GEN, STR, KAN, TET, NA, SXT, CHL, ERY, MEM	0.67	S	S	S
M5	Milk	1/2c	+	+	−	−	+	+	+	GEN, STR, KAN, NA	0.27	N	N	W
C6	Chicken	4b	+	+	−	−	+	+	+	CTX, GEN, STR, KAN, TET, NA, SXT, CHL, ERY, MEM	0.67	N	N	N
C7	Chicken	4b	+	+	+	+	+	+	+	AMC, CTX, CEF, GEN, STR, KAN, TET, NA, SXT, CHL, ERY, MEM	0.8	S	S	S
C8	Chicken	4b	+	+	+	+	+	+	+	GEN, STR, KAN, NA, SXT, MEM	0.4	S	S	S
C9	Chicken	1/2a	+	+	+	+	+	+	+	AMC, CTX, CEF, GEN, STR, KAN, TET, NA, SXT, CHL, ERY, MEM	0.8	S	S	S
C10	Chicken	1/2a	+	+	−	−	+	+	+	GEN, STR, KAN, NA	0.27	N	W	W
C11	Chicken	1/2c	+	+	−	−	+	+	+	CEF, STR, KAN, NA	0.27	N	N	W
C12	Chicken	1/2c	+	+	−	−	+	+	+	GEN, STR, KAN, NA	0.27	N	N	W
C13	Chicken	4b	+	+	+	−	+	+	+	GEN, STR, KAN, NA, SXT, CHL, ERY, MEM	0.53	W	M	M
C14	Chicken	1/2a	+	+	−	−	+	+	+	GEN, STR, KAN, TET, NA, SXT, MEM	0.47	W	M	M
C15	Chicken	1/2b	+	+	−	−	+	+	+	GEN, STR, KAN, NA, MEM	0.33	W	M	M
C16	Chicken	1/2b	+	+	−	−	+	+	+	GEN, STR, KAN, NA, MEM	0.33	W	M	M
C17	Chicken	1/2a	+	+	−	−	−	+	+	GEN, STR, KAN, NA	0.27	N	W	W
W18	Women	4b	+	+	+	+	+	+	+	AMC, CTX, CEF, GEN, STR, KAN, TET, NA, SXT, CHL, ERY, MEM	0.8	M	M	S
W19	Women	4b	+	+	+	−	+	+	+	GEN, STR, KAN, NA, MEM	0.33	W	M	M
W20	Women	1/2a	+	+	+	+	+	+	+	GEN, STR, KAN, TET, NA, SXT, CHL, ERY, MEM	0.6	M	M	S
W21	Women	1/2a	+	+	+	−	+	+	+	GEN, STR, KAN, NA, SXT, ERY, MEM	0.47	M	M	S
W22	Women	1/2c	+	+	−	−	+	+	+	GEN, STR, KAN, NA, SXT, CHL, MEM	0.47	N	W	M
W23	Women	1/2a	+	+	−	−	+	+	+	GEN, STR, KAN, NA	0.27	N	N	N
W24	Women	1/2b	+	+	−	−	+	+	+	GEN, STR, KAN, NA, SXT	0.33	N	N	N
W25	Women	1/2c	+	+	−	−	+	+	+	GEN, STR, KAN, NA, SXT	0.33	N	N	N
W26	Women	1/2b	+	+	−	−	+	+	+	GEN, STR, KAN, NA, SXT	0.33	N	N	N

1 +, virulence genes positive; −, virulence genes negative.

2 AMP, ampicillin; SAM, ampicillin/sulbactam; AMC, amoxicillin-clavulanate; CTX, cefotaxime; CEF, cephalothin; GEN, gentamicin; STR, streptomycin; KAN, kanamycin; TET, tetracycline; CIP, ciprofloxacin; NA, nalidix acid; SXT, trimethoprim-sulfamethoxazole; CHL, chloramphenicol; ERY, erythromycin; MEM, meropenem.

3 MAR, multiple antibiotic resistance index.

4 N, negative biofilm forming; W, weak biofilm forming; M, moderate biofilm forming; S, strong biofilm forming.

### 3.3. Antimicrobial susceptibility test

**Figure 1:**
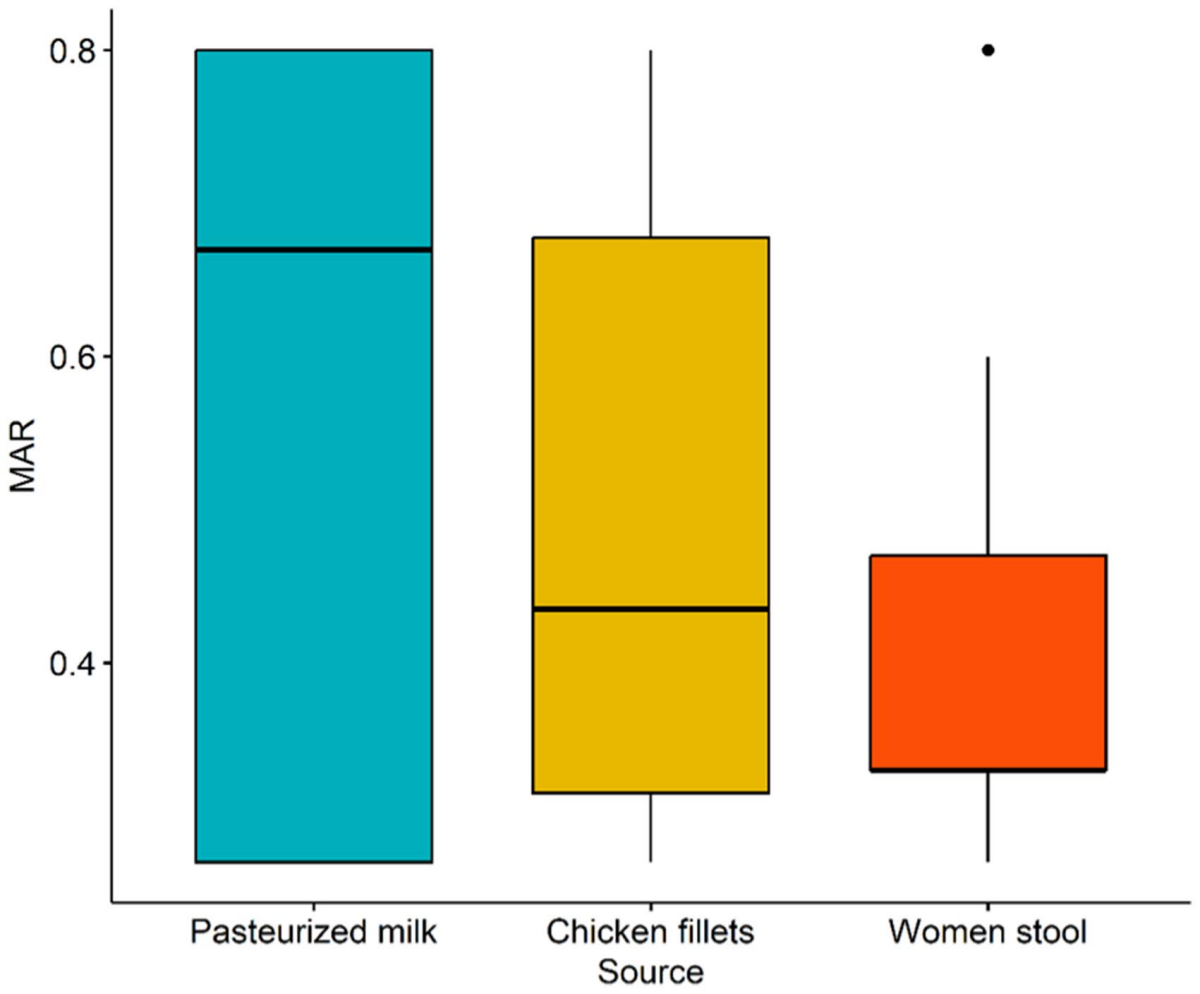
Box plot of multiple antibiotic resistance (MAR) index of *L. monocytogenes* isolates recovered from pasteurized milk, chicken fillets and stool of pregnant women.

Table 2 shows the resistance patterns of *L. monocytogenes* isolates to the 15 tested antimicrobials. All isolates were resistant to STR, KAN, and NA (100%), with GEN resistance observed in 92.3% of isolates. Nevertheless, all isolates were susceptible to AMP and SAM (Table 3). Multidrug resistance was observed in 20 (76.9%) *L. monocytogenes* isolates. MDR was observed in 20 (76.9%) of *L. monocytogenes* isolates. The mean MAR index, which ranged from 0.27 to 0.80, was 0.47. The *L. monocytogenes* isolated from pasteurized milk had the highest MAR index, followed by those from chicken fillets, but the MAR index did not differ significantly (*p*-value = 0.8204) among isolates from pasteurized milk, chicken fillets, and stool samples collected from pregnant women (Figure 1).

**Table 3:** Results of antimicrobial resistance of *L. monocytogenes* isolates.

Rank^1^	Antimicrobial class	Antibiotic	Number (%) of *L. monocytogenes*^2^		
			R	I	S
II	Penicillins	Ampicillin	0 (0.0)	0 (0.0)	26 (100)
		Ampicillin/sulbactam	0 (0.0)	0 (0.0)	26 (100)
II	Cephalosporine	Amoxicillin-clavulanate	5 (19.2)	0 (0.0)	21 (80.8)
		Cefotaxime	7 (26.9)	3 (11.5)	16 (61.5)
		Cephalothin	6 (23.1)	3 (11.5)	17 (65.4)
I	Aminoglycosides	Gentamicin	24 (92.3)	2 (7.7)	0 (0)
		Streptomycin	26 (100)	0 (0)	0 (0)
		Kanamycin	26 (100)	0 (0)	0 (0)
II	Tetracyclines	Tetracycline	10 (38.5)	0 (0.0)	16 (61.5)
I	Quinolones	Ciprofloxacin	0 (0.0)	2 (12.5)	14 (87.5)
		Nalidix Acid	26 (100)	0 (0.0)	0 (0.0)
II	Sulphonamides	Trimethoprim-sulfamethoxazole	16 (61.5)	0 (0.0)	10 (38.5)
II	Phenicols	Chloramphenicol	10 (38.5)	0 (0.0)	16 (61.5)
I	Macrolides	Erythromycin	10 (38.5)	0 (0.0)	16 (61.5)
I	Carbapenem	Meropenem	16 (61.5)	0 (0.0)	10 (38.5)

1 Rank of antimicrobial agents is based on World Health Organization’s categorization of critical importance in human drugs. Rank I, critically important; rank II, highly important.

2 R, resistant; I, intermediate; S, sensitive.

### 3.4. Biofilm formation

The biofilm formation ability of 26 *L. monocytogenes* isolates was evaluated at various incubation temperatures (Table 2). At 4°C, 25°C, and 37°C, 12 (46.2%), 8 (30.8%), and 5 (19.2%) isolates, respectively, were non-biofilm producers. Additionally, 6 (23.1%) isolates demonstrated strong biofilm formation ability at both 4°C and 25°C. The number of strong biofilm-producing isolates increased to 9 (34.6%) when incubated at 37°C.

### 3.5. Effect of *Lactobacillus plantarum* on *Listeria monocytogenes*

**Figure 2:**
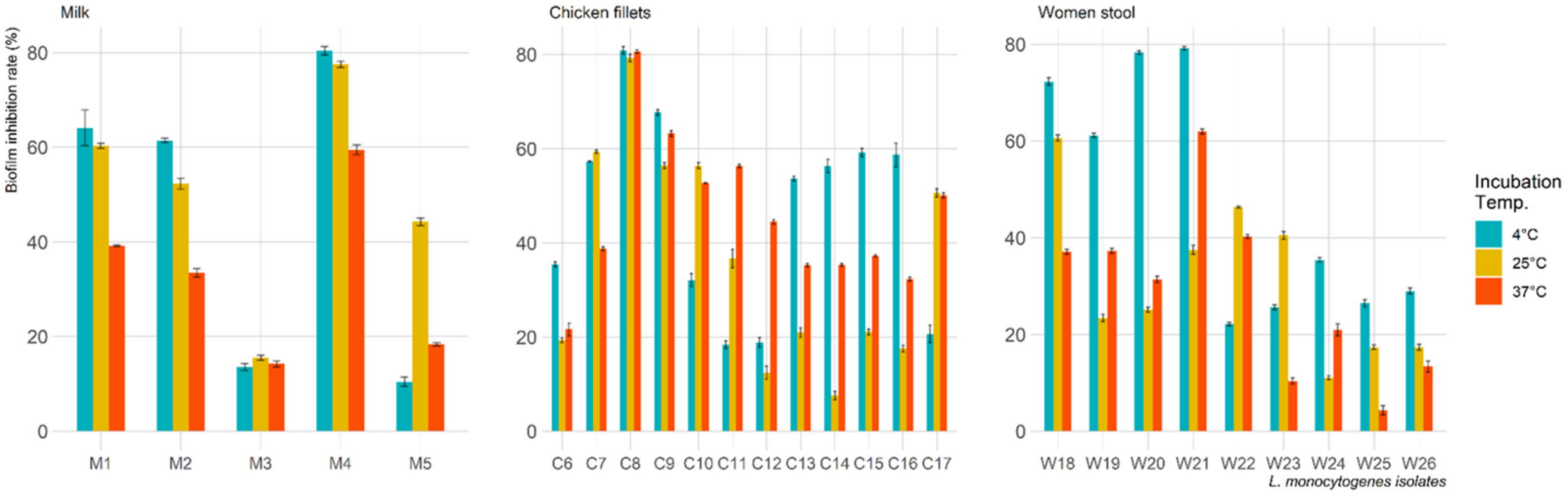
Biofilm inhibition rate of *L. plantarum* cell free supernatant against *L. monocytogenes* isolates at various incubation temperature.

Among the 26 *L. monocytogenes* isolates, 53.8, 69.2, and 80.8% were found to be biofilm producers at 4°C, 25°C, and 37°C, respectively. However, treatment with *L. plantarum* CFS resulted in a decrease in the number of biofilm-producing isolates to 15.4, 42.3, and 53.8% at 4°C, 25°C, and 37°C, respectively. Figure 2 displays the biofilm inhibition rate of *L. plantarum* CFS against *L. monocytogenes* isolated from pasteurized milk, chicken fillets, and the stool of pregnant women and incubated at 4°C, 25°C, and 37°C. The highest biofilm inhibition rate observed at 4°C, 25°C and 37°C were 81.1, 79.6 and 80.8%, respectively.

Table 4 shows the antibacterial activity of *L. plantarum* CFS against six virulent, MDR, and biofilm-forming *L. monocytogenes* strains isolated from pasteurized milk, chicken fillets, and pregnant women’s stools. The positive control used was AMP, which was sensitive to all isolates. At a concentration of 5 μg/mL of *L. plantarum* CFS, none of the *L. monocytogenes* isolates exhibited an inhibition zone. However, an inhibition zone was observed against *L. monocytogenes* strains isolated from pasteurized milk and pregnant women’s stools when using a concentration of 10 μg/mL. The inhibition zone significantly increased as the *L. plantarum* CFS concentration was increased from 10 to 100 μg/mL. Additionally, the results indicated that the MIC value of *L. plantarum* CFS required to inhibit visible growth of *L. monocytogenes* was 10 μg/mL.

**Table 4:** Antibacterial activity of cell free supernatant of *L. plantarum* against *L. monocytogenes* isolates recovered from pasteurized milk, chicken fillets and stool of pregnant women.

Probiotics	Concentration (μg/mL)	Inhibition zone diameter (mm) of *L. monocytogenes* isolates					
		Pasteurized milk		Chicken fillets		Pregnant women stool	
		M1	M2	C7	C9	W18	W20
Ampicillin	10	18.0 ± 0.05	23.0 ± 0.05	21.2 ± 0.26	22.0 ± 0.07	19.1 ± 0.32	21.2 ± 0.38
*L. plantarum*	5	0.0	0.0	0.0	0.0	0.0	0.0
	10	27.6 ± 0.2	0.0	0.0	0.0	19.2 ± 0.36	0.0
	20	36.0 ± 1.05	23.6 ± 0.06	18.0 ± 0.45	28.1 ± 0.42	29.9 ± 0.83	0.0
	30	41.0 ± 0.02	28.7 ± 0.06	25.1 ± 0.09	31.3 ± 0.20	33.6 ± 0.20	16.7 ± 0.06
	40	49.0 ± 0.06	38.2 ± 1.05	30.0 ± 0.95	40.3 ± 0.15	38.3 ± 0.10	23.7 ± 0.15
	50	53.7 ± 0.10	41.1 ± 1.01	34.1 ± 1.10	44.3 ± 0.20	45.0 ± 0.05	26.3 ± 0.61
	70	54.9 ± 0.40	44.0 ± 0.26	36.4 ± 0.35	47.1 ± 0.15	46.4 ± 0.12	27.0 ± 0.55
	100	55.13 ± 0.80	44.2 ± 1.30	36.4 ± 0.15	47.2 ± 0.40	46.3 ± 0.20	27.0 ± 0.35

**Figure 3:**
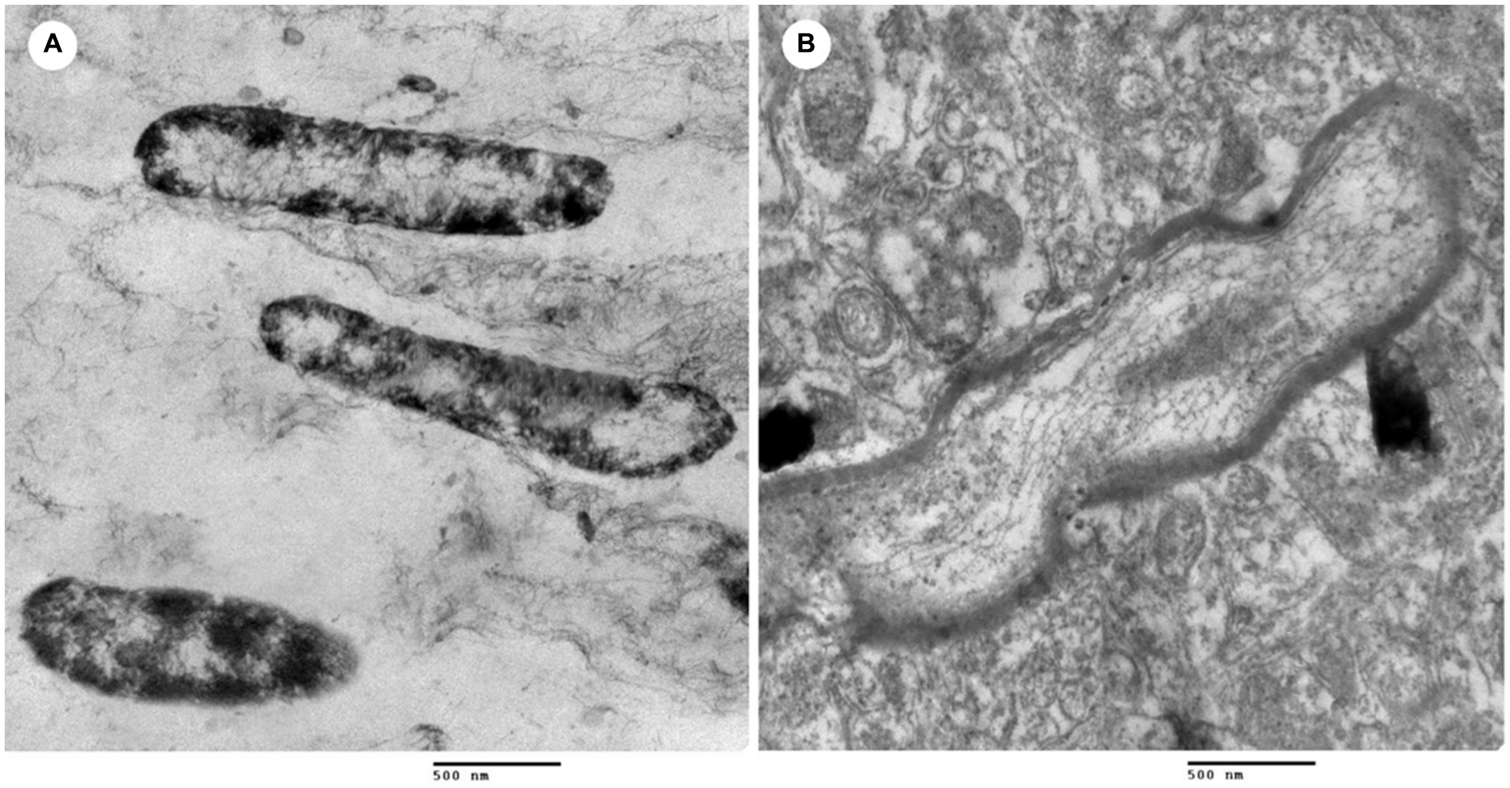
Transmission electron microscope of *L. monocytogenes*. **(A)** *L. monocytogenes* (control) and **(B)** *L. monocytogenes* treated with cell free supernatant of *L. plantarum*.

The TEM observed morphological and intracellular structural changes of *L. monocytogenes* treated with *L. plantarum* CFS (Figure 3). Figure 3A shows untreated *L. monocytogenes* cells with well-defined, short rod shapes and uniformly distributed cytoplasm. In contrast, Figure 3B displays *L. monocytogenes* cells treated with 10 μg/mL of *L. plantarum* CFS, where the cell membrane was damaged and the intracellular contents leaked out.

## 4. Discussion

*Listeria* spp. is a significant public health pathogen that causes serious illnesses and sporadic foodborne outbreaks (Zunabovic et al., 2011). It is commonly found in dairy and meat products and has deleterious effects not only on public health but also on the global economy. In this study, *Listeria* spp. was isolated from pasteurized milk, chicken fillets, and pregnant women’s stools. Chicken fillets had the highest isolation rate of *Listeria* spp., followed by pregnant women’s stool samples and pasteurized milk. Among the *Listeria* spp. identified, *L. innoua* was the most prevalent, followed by *L. monocytogenes*. Several studies have also identified *Listeria* spp. in chicken meat products (Matle et al., 2019), pasteurized milk (Moura et al., 1993), and pregnant women (El-Naenaeey et al., 2019). However, the isolation rate in our study was similar to the rate previously reported in chicken fillets from Egypt (56%; El-Malek et al., 2010), and higher than rates reported in pasteurized milk from Brazil (0.9%; Moura et al., 1993), and pregnant women from northern Ethiopia (8.5%; Welekidan et al., 2019). The variation in the isolation rate of *Listeria* spp. between studies could be due to sample sizes and isolation methods.

*Listeria monocytogenes* ranks as the third major foodborne pathogen in terms of economic burden in the United States (Hoffmann et al., 2015). In this study, *L. monocytogenes* was found in 20% of pasteurized milk, 30.6% of chicken fillets, and 23.1% of pregnant women’s stool samples. These rates were higher than the 12% reported in pasteurized milk (Saleh et al., 2021) and 4% in pregnant women in Egypt (El-Naenaeey et al., 2019), but lower than the 44% found in chicken fillets from Egypt (El-Malek et al., 2010). The presence of *L. monocytogenes* in the samples suggests possible inadequate hygiene practices, cross-contamination, improper handling, or inadequate storage temperatures (Letchumanan et al., 2018). Factors such as poor-quality milk, unsanitary manufacturing conditions, substandard materials, and inadequate water for utensil washing, as well as unclean hands of workers, could potentially contribute to bacterial contamination of dairy and meat products during manufacturing and post-manufacturing stages (Kulshrestha, 1990).

*Listeria monocytogenes* pathogenicity is associated with determination of virulence genes. The *Listeria* Pathogenicity Island 1 (LIPI-1) virulence genes have a significant role in *L. monocytogenes* intracellular life cycle and cellular infection (Vázquez-Boland et al., 2001). LIPI-1 virulence genes (*hlyA*, *actA*, *inlC* and *inlJ*) were identified in all *L. monocytogenes* isolates recovered in this study. However, these genes (*plcB*, *iap*, and *inlA*) were detected in only 38.5, 30.8, and 96.2% of the isolates, respectively. These findings are consistent with previous studies that have detected virulence genes in milk products (Osman et al., 2016; Tahoun et al., 2017), raw meat, and meat products (Oliveira et al., 2018), as well as in clinical and food isolates associated with major listeriosis outbreaks (Todd and Notermans, 2011; Leong et al., 2014). The presence or absence of LIPI-1 virulence gene can contribute to variations in the virulence potential of different *L. monocytogenes* isolates (Vázquez-Boland et al., 2001). Thus, prevalence of *L. monocytogenes* carrying virulence genes found in this study indicates that these pathogens could pose a significant risk to public health.

The present study identified four *L. monocytogenes* serotypes (1/2a, 1/2b, 1/2c, and 4b), with serotypes 1/2a and 4b being the most prevalent (30.8%), followed by 1/2b and 1/2c (19.2%). The distribution of these serotypes is predictable since they are frequently isolated from food samples (Montero et al., 2015). Previous studies reported similar trends but with a higher proportion. Muraoka, Gay (Muraoka et al., 2003) reported that serotypes 1/2a and 4b were the most frequently observed strains isolated from bulk milk in the Pacific Northwest. Serotypes 1/2a and 4b of dairy and meat products have been associated with several listeriosis outbreaks (Pan et al., 2009). Furthermore, serotypes 4b, 1/2b, and 1/2a are predominant strains associated with foodborne human listeriosis (Soni et al., 2014; Chen et al., 2017), suggesting that these isolates may exhibit pathogenicity against consumers (Orsi et al., 2011).

The antimicrobial susceptibility of *L. monocytogenes* isolates was variable in the present study. All isolates were resistant to aminoglycosides (GEN, STR, and KAN) and quinolones (NA), and susceptible to penicillins (PEN and AMC), which is consistent with a previous study in Egypt, except for a higher proportion of resistance to SXT (Osman et al., 2021). Moreover, a high proportion of *L. monocytogenes* isolates in this study showed resistance to CTX, CEF, TET, and CHL, consistent with previous studies in Egypt (Tahoun et al., 2017) and China (Chen et al., 2019), suggesting that antimicrobial misuse may accelerate the emergence of AMR in *L. monocytogenes*. Notably, all *L. monocytogenes* isolates in this study were susceptible to penicillins, which are recommended for human listeriosis treatment (Olaimat et al., 2018). However, the resistance to ERY is concerning as it is the drug of choice for treating listeriosis in pregnant women.

MDR *L. monocytogenes* have been isolated from various sources including food (Conter et al., 2009). In this study, 79.6% of the *L. monocytogenes* isolates exhibited MDR, which is lower than the 88% previously reported in Egypt (Tahoun et al., 2017), but higher than the prevalence of MDR *L. monocytogenes* found in dairy and meat products in other studies (Doyle et al., 2013; Kevenk and Terzi, 2016). The average MAR index was 0.47, which is also higher than the 0.34 previously reported in *L. monocytogenes* isolates from Egypt (Tahoun et al., 2017). Furthermore, all isolates had MAR index greater than 0.2, indicating that there was antimicrobial misuse and growing resistance among *L. monocytogenes* isolates.

Biofilm formation in food processing environments poses a serious safety problem for processed food and is difficult to remove (Li et al., 2018). In the present study, 80.8% of *L. monocytogenes* isolates showed the ability to form biofilm at 37°C, and of these isolates, 34.6% showed strong biofilm-forming ability. The biofilm-forming potential of *L. monocytogenes* is influenced by the presence of virulence genes (Price et al., 2018) and antimicrobial resistance (Kayode and Okoh, 2022). MDR strains were associated with strong biofilm-forming ability, which might be attributed to the higher tolerance of these isolates to disinfectants and antimicrobials (Doulgeraki et al., 2017).

Our study determined the anti-biofilm effects of *L. plantarum* CSF on *L. monocytogenes* at different concentrations and incubation times. The results indicate that *L. plantarum* CSF can effectively inhibit biofilm formation of *L. monocytogenes*, with the highest inhibition rate (81.1%) observed at 4°C. Previous research has shown that probiotics, including LAB, can prevent biofilm formation by foodborne pathogens such as *L. monocytogenes* (Hossain et al., 2017; Monteagudo-Mera et al., 2019; Lee et al., 2022). LAB contain various anti-biofilm agents, such as hydrogen peroxide, oxygen metabolites, exopolysaccharides, bacteriocin, and saturated fatty acids acting as biosurfactants (Moradi et al., 2019).

All tested *L. monocytogenes* were inhibited by *L. plantarum* CFS with different concentration. It was previously reported that *L. plantarum* and *L. rhamnous* could inhibit *L. monocytogenes*, *E. coli* and *Salmonella typhimurium* colonization (Lau and Chye, 2018; Lee et al., 2022). *L. plantarum* at different concentration had higher anti-bacterial activity than AMP (10 μg/mL) against *L. monocytogenes*. The MIC of *L. plantarum* CFS was determined at 10 μg/mL with diameters inhibition zones ranged from 19.2 ± 0.36 to 27.6 ± 0.2. Therefore, *L. plantarum* could be considered to have anti-bacterial activity against *L. monocytogenes* in this study. In the present study, *L. plantarum* CFS inhibited all tested *L. monocytogenes* at different concentrations. Previous studies have shown that *L. plantarum* and *L. rhamnosus* can prevent colonization of *L. monocytogenes*, *E. coli*, and *Salmonella typhimurium* (Lau and Chye, 2018; Lee et al., 2022). Furthermore, *L. plantarum* demonstrated higher anti-bacterial activity against *L. monocytogenes* than AMP (10 μg/mL) at different concentrations. These findings suggest that *L. plantarum* possesses anti-bacterial activity against *L. monocytogenes*.

The changes in the morphology and intercellular structure of *L. monocytogenes* after treated with *L. plantarum* have been investigated under TEM. Results showed that the treated *L. monocytogenes* experienced deterioration of cell membranes, cell swelling, and vacuole formation, ultimately leading to cell lysis. These observations are consistent with findings reported by Chlebowska-Smigiel, Gniewosz (Chlebowska-Smigiel et al., 2017), who attributed the antibacterial activity of *L. plantarum* to the release of antimicrobial metabolites and inhibitory compounds that surround *L. monocytogenes*. *L. plantarum* has also been found to initiate the formation of pores in the bacterial cell membrane, causing leakage of essential molecules and ions that ultimately results in cell death (Cotter et al., 2013; Osman et al., 2021). A previous study has reported that the inhibitory effect of *L. plantarum* CFS is attributed to the release of bacteriocin and organic acids (Moradi et al., 2019). Bacteriocin’s positively charged amino acid residues facilitate pore formation and exert electrostatic forces on cell membranes, leading to the leakage of cell electrolytes and subsequent cell lysis (Moradi et al., 2019). However, the organic acids present in *L. plantarum* CFS reduce the pH, creating an acidic environment that inhibits the growth of pathogenic bacteria (Enan et al., 2014; Osman et al., 2021). Moreover, *L. plantarum* CFS can hinder biofilm formation of *L. monocytogenes* through nutrient emulation and adhesion area intervention (Hossain et al., 2017), or by producing antimicrobial compounds that react with the pathogen or biofilm model compounds, as reported in previous literature (Kim et al., 2021).

## 5. Conclusion

The present study found *Listeria* spp. in pasteurized milk, chicken fillets, and stool samples from pregnant women in Sharkia Governorate, Egypt, particularly, *L. innoua* and *L. monocytogenes*. The study also revealed the emergence of virulent MDR *L. monocytogenes* with strong biofilm formation abilities in food products in Egypt, posing significant risks to food safety. Although *L. plantarum* exhibited potential antibacterial and anti-biofilm effects against *L. monocytogenes* isolates, further research is necessary to explore its full probiotic potential. Lastly, it is crucial to monitor the prevalence and antimicrobial resistance profile of *L. monocytogenes* in dairy and meat products to enhance their safety.

## Data availability statement

The original contributions presented in the study are included in the article/Supplementary material, further inquiries can be directed to the corresponding author.

## Ethics statement

The animal study was reviewed and approved by the Institutional Animal Care and Use Committee (IACUC) of Zagazig University (Ref. No.: ZU-IACUC/2/F/1/2023).

## Author contributions

RAb, A-RA-M, MS, AT, AA, and NE-G contributed to the conception and design of the study. RAb and NE-G carried out the practical parts. RAb, IE, and NE-G performed the statistical analysis and interpretation of the results and wrote the manuscript’s initial draft. A-RA-M, MS, AT, RAl, EM, and AA edited and critical appraisal of the manuscript. All authors reviewed and approved the final manuscript.

## Conflict of interest

The authors declare that the research was conducted in the absence of any commercial or financial relationships that could be construed as a potential conflict of interest.

## Publisher’s note

All claims expressed in this article are solely those of the authors and do not necessarily represent those of their affiliated organizations, or those of the publisher, the editors and the reviewers. Any product that may be evaluated in this article, or claim that may be made by its manufacturer, is not guaranteed or endorsed by the publisher.
