# Supplementary material for: Antibacterial and anti-biofilm activities of probiotic Lactobacillus plantarum against Listeria monocytogenes isolated from milk, chicken and pregnant women
Source: Front Microbiol. 2023 Jul 19;14:1201201. doi: 10.3389/fmicb.2023.1201201 (PMC10394229; doi:10.3389/fmicb.2023.1201201)

Supplementary Material

Antibacterial and anti-biofilm activities of probiotic *Lactobacillus plantarum* against *Listeria monocytogenes* isolated from milk, chicken and pregnant women

**Rasha M.M. Abou Elez^1†^, Ibrahim Elsohaby^2,3,4,*†^, Abdul-Raouf Al-Mohammadi^5^, Marwa M. Seliem^1^, Asmaa B.M.B. Tahoun^6^, Amira I. Abousaty^7^, Reem M. Algendy^6^, Eman A.A. Mohamed^8^ and Nashwa El-Gazzar^7†^**

^1^Department of Zoonoses, Faculty of Veterinary Medicine, Zagazig University, Zagazig City 44511, Sharkia, Egypt

^2^Department of Infectious Diseases and Public Health, Jockey Club College of Veterinary Medicine and Life Sciences, City University of Hong Kong, Hong Kong SAR, China

^3^Centre for Applied One Health Research and Policy Advice (OHRP), City University of Hong Kong, Hong Kong SAR, China

^4^Department of Animal Medicine, Faculty of Veterinary Medicine, Zagazig University, Zagazig City 44511, Sharkia, Egypt

^5^Department of Science, King Khalid Military Academy, Riyadh 11495, Saudi Arabia

^6^Department of Food Hygiene, Safety and Technology, Faculty of Veterinary Medicine, Zagazig University, Zagazig City 44511, Sharkia, Egypt

^7^Department of Botany and Microbiology, Faculty of Science, Zagazig University, 44519, Egypt

^8^Department of Microbiology, Faculty of Veterinary Medicine, Zagazig University, Zagazig City 44511, Sharkia, Egypt

^†^ These authors contributed equally to this work.

*** Correspondence:**Ibrahim Elsohaby
[ielsohab@cityu.edu.hk](mailto:ielsohab@cityu.edu.hk)

# Supplementary Tables and Figures

**Table S1**. Nucleotide sequences and annealing temperature of *L. monocytogenes* genes primers.

| **Primers use and target gene** | **Nucleotide sequence (5′→3′)** | **Amplicon size (bp)** | **Annealing temperature** | **Reference** |
| --- | --- | --- | --- | --- |
| *16S rRNA* | F: CCT TTG ACC ACT CTG GAG ACA GAG C | 553 | 60°C | (25) |
|  | R: AAG GAG GTG ATC CAA CCG CAC CTT C |  |  |  |
| *hly* | F: ACGCAGTAAATACATTAGTG | 372 | 54°C | (26) |
|  | R: AATAAACTTGACGGCCATAC |  |  |  |
| *actA* | F: TGCATTACGATTAACCCCGACA | 431 |  |  |
|  | R: AGGCTTTCAAGCTCACTATCCG |  |  |  |
| *plcB* | F: AGTGTTCTAGTCTTTCCGG | 792 |  |  |
|  | R: ACCTGCCAAAGTTTGCTGT |  |  |  |
| *iap* | F: TTTGCTAAAGCGGGTATCTC | 205 |  |  |
|  | R: AGCCGTGGATGTTATCGTAT |  |  |  |
| *inlA* | F: ACG AGT AAC GGG ACA AAT GC | 800 | 55°C | (11) |
|  | R: CCC GAC AGT GGT GCT AGA TT |  |  |  |
| *inlC* | F: AATTCCCACAGGACACAACC | 517 |  |  |
|  | R: CGGGAATGCAATTTTTCACTA |  |  |  |
| *inlJ* | F: TGTAACCCCGCTTACACAGTT | 238 |  |  |
|  | R: AGCGGCTTGGCAGTCTAATA |  |  |  |
| *lmo1118* | F: AGGGGTCTTAAATCCTGGAA | 906 | 53°C | (27) |
|  | R: CGGCTTGTTCGGCATACTTA |  |  |  |
| *ORF2819* | F: AGCAAAATGCCAAAACTCGT | 417 |  |  |
|  | R: CATCACTAAAGCCTCCCATTG |  |  |  |
| *ORF2110* | F: AGTGGACAATTGATTGGTGAA | 597 |  |  |
|  | R: CATCCATCCCTTACTTTGGAC |  |  |  |

**Figure S1.** The growth kinetic curve of *L. monocytogenes* treated with *L. plantarum* cell-free supernatant (CFS).


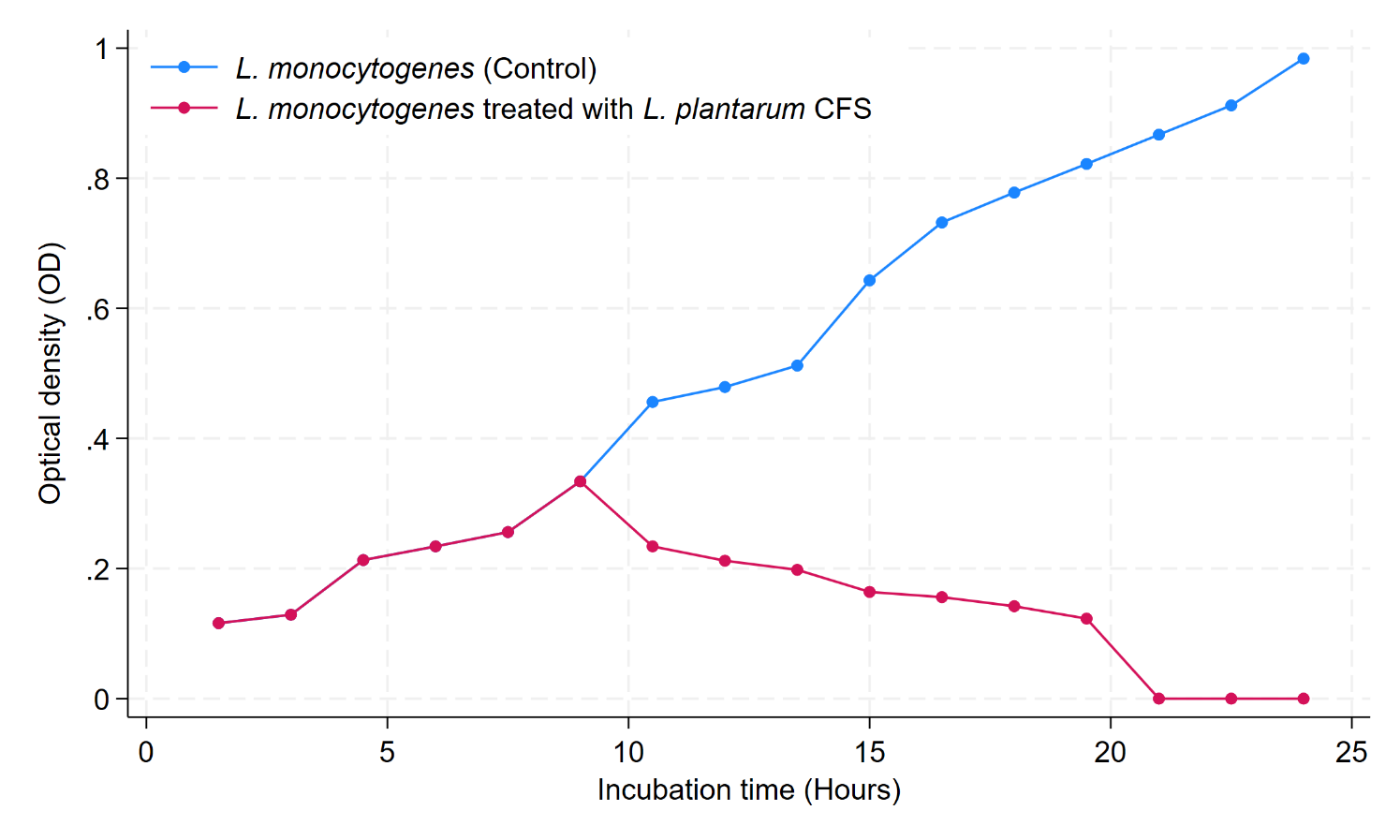

Supplement: Supplementary file 1 [file Data_Sheet_1.docx]
